# Patient and Public Involvement and Engagement in Pediatric Health Research: A Systematic Review

**DOI:** 10.1111/hex.70862

**Published:** 2026-09-04

**Authors:** Veronika Stadler, Rebeca Mozun, Jannik Strohm, SwissPedHealth consortium, Chloé Caruso, Klara M. Posfay‐Barbe, Cornelia Hagmann

**Affiliations:** ^1^ Department of Intensive Care and Neonatology University Children's Hospital Zurich and Children's Research Center, University of Zurich Zurich Switzerland; ^2^ Faculty of Medicine University of Zurich Zurich Switzerland; ^3^ See list of the SwissPedHealth consortium under Collaborators; ^4^ Division of General Pediatrics, Department of Women, Child and Adolescent, University Hospitals of Geneva & Faculty of Medicine University of Geneva Geneva Switzerland

**Keywords:** adolescent, biomedical research, child, community‐based participatory research, health services research, patient participation, pediatrics

## Abstract

**Background:**

Patient and public involvement and engagement (PPIE) can increase the relevance and efficiency of research projects. An overview of PPIE approaches and implementation in pediatric research studies is needed to facilitate learning from others' experiences.

**Objective:**

We aimed to systematically review practices in PPIE across all pediatric health research disciplines regarding characteristics and recruitment of PPIE participants, timepoints and methods used for PPIE, levels of involvement, benefits and barriers of PPIE.

**Search Strategy:**

We searched Pubmed, EMBASE, Cochrane and PsycInfo using a comprehensive set of terms based on the concepts ‘Patient and Public Involvement,’ ‘Health Research’ and ‘Pediatrics.’

**Inclusion Criteria:**

We included original research articles describing PPIE implementation in pediatric health research published in English or German between 01/2003–10/2024.

**Data Extraction and Synthesis:**

Data was extracted using predefined categories and synthesized by narrative summary and thematic synthesis. PPIE reporting quality was assessed using the GRIPP2 short form checklist.

**Main Results:**

Out of 1910 references, we included 37 original research articles, representing 35 studies. PPIE participants were mostly children, adolescents or caregivers involved in all research stages, especially in study design (89%) and recruitment (51%). Key positive impacts of PPIE on research included enhanced recruitment and retention rates and personal benefits for PPIE participants. Barriers to PPIE were financial and time resources required and challenges in recruiting representative PPIE participants. The level of involvement and PPIE reporting quality varied highly between studies.

**Discussion:**

Common benefits and barriers of PPIE exist across pediatric research disciplines. Reporting quality varied highly between studies.

**Conclusions:**

PPIE is valuable in pediatric health research. Adherence to guidelines for conducting and reporting PPIE is important to enhance mutual learning.

**Patient or Public Contribution:**

PPIE input contributed to the understandability of the lay summary. The findings of this review, together with parent and public input, will inform guidelines for future PPIE activities at the authors' institutions.

## 1 Introduction

Patients' or public's lived experience and lay views can support the development of more effective, acceptable and relevant health research projects [3]. This approach, named patient and public involvement and engagement (PPIE), is defined as ‘research being carried out “with” or “by” members of the public rather than “to,” “about” or “for” them’ [4]. There is a clear difference between being a PPIE participant and being a study participant. The latter refers to patients taking part in a research project as study subjects, for example by providing samples or data. In contrast, PPIE means that patients or the public are actively involved as research partners by sharing their lived experience or lay views [5]. PPIE participants can contribute to each study phase, such as defining relevant research questions and study outcomes, or dissemination of findings. The aim of PPIE is to improve the relevance and quality of research. In pediatric health research, it is reasonable that children, young people and families play a major role in PPIE [3], since they are the ones who should ultimately benefit from research outcomes. It is particularly crucial to capture the perspectives of children and young people, rather than having their views represented exclusively by caregivers. Thereby, guidance on key considerations for involvement of children and young people can be used by researchers [6]. PPIE participants can contribute in different levels of involvement, as represented in the Montreal Model [7]. The first level is information, where there is little dialogue, but the patient is informed about research. At the second level, consultation, a researcher obtains the patients' view, and the patient can ask questions. The next level is involvement, in which the patient has a more active role by contributing to decision‐making. At the highest level of involvement, the partnership level, researchers and patients collaborate as equal partners in research. The level of involvement must be defined individually for each project. Several guidelines for conducting and reporting PPIE in research are available. For example, the National Institute for Health and Care Research (NIHR) provides general guidance on PPIE in their briefing notes [4] and shares practical and essential information on involving children and young people as advisors in research [8]. The GRIPP2 reporting checklists [9] provide an international, evidence based, and consensus informed framework for reporting PPIE in research. The guidelines are important to increase the quality, consistency and transparency of PPIE knowledge and evidence.

There are reviews summarizing PPIE activities in various disciplines of health research focusing on adults [10, 11, 12]. For instance, Domecq et al. [13] included in their systematic review on patient engagement in research mainly studies reporting PPIE adult health research. Their summary of practical PPIE examples, focusing on PPIE participant recruitment, PPIE methods as well as barriers and impacts can support researchers to implement PPIE in their own projects. In contrast, comparable reviews in pediatric health research, defined as research studies in which the majority of study participants is younger than 18 years, remain limited. Existing reviews have examined the involvement of children and young people up to 26 of age as PPIE participants in health research, but not the involvement of caregivers [14, 15, 16, 17, 18, 19, 20, 21, 22, 23]. Some reviews have explored PPIE within specific pediatric health research areas, such as mental health research, critical care research, or health services research [15, 22, 24, 25], or have focused primarily on describing the impact of PPIE [3]. There is a lack of comprehensive reviews capturing the full range of PPIE approaches implemented in pediatric health research, regardless of PPIE participant age, disease area or reported impact outcomes. To address this gap, our systematic review captures how PPIE participants in pediatric health research are recruited, which PPIE approaches are used, the positive impact of PPIE on research and PPIE participants, as well as potential barriers and negative impacts. This systematic review aims to identify original research articles that describe practical examples of PPIE in pediatric health research to facilitate the implementation of PPIE for researchers in future projects.

## 2 Methods

This is a systematic review of original research articles reporting PPIE in pediatric health research. The protocol was registered with OSF (10.17605/OSF. IO/WPFUM) and PROSPERO (CRD420251035067). This systematic review is reported according to PRISMA reporting guidelines. We provide a lay summary that can be used for dissemination in Appendix 1. To maintain terminological consistency, the term ‘involvement’ is used in this systematic review to denote both involvement and engagement.

### 2.1 Research Questions

The aim of this systematic review is to provide an overview of practices in PPIE in pediatric health research. We focused on five key research questions:

1. What are the characteristics of PPIE participants and how are they identified and recruited?
2. At which timepoints are PPIE participants involved in pediatric health research?
3. Which methods are used to involve PPIE participants in pediatric health research and what is the level of involvement?
4. What are the observed benefits and the positive impact of PPIE in pediatric health research?
5. What are the barriers to and negative impact of PPIE in pediatric health research?

### 2.2 Database Search Strategy

The literature search was conducted to identify references published from 01/2003 to 10/2024. The search strategy was developed based on the concepts ‘Patient and Public Involvement,’ ‘Health Research’ and ‘Pediatrics.’ A comprehensive set of search terms was developed and iteratively refined by the research team. Boolean operators, as well as truncation and proximity operators were applied to enhance the sensitivity of the search strategy. This process was supported by a liaison librarian. Database searches were carried out using Pubmed, EMBASE, Cochrane and PsycInfo. The reference lists of the reviews and articles included in the review were screened to identify further relevant papers. Search strategies are available in Appendix 2.

### 2.3 Inclusion and Exclusion Criteria

We included original research articles of studies that described the involvement of patients or the public in the design, conduct, or dissemination of a study in pediatric health research. We defined pediatric health research as research studies in which the majority of study participants is younger than 18 years. Patients and the public are people who shared their lay, non‐professional experience or opinion in a research project, such as parents, caregivers, or children. We did not set restrictions regarding sample size, study design or study country and we included articles published in English or German. The protocol planned to include systematic reviews. We made a post‐hoc decision to exclude them to focus the synthesis on original research articles. Previous systematic reviews are referenced in the Introduction and Discussion.

We excluded original research articles in which PPIE activities were carried out for projects that did not have a primary focus on pediatrics or health research. In addition, we excluded non‐original research articles, such as conference abstracts, commentaries, theses, editorials, and reviews. We excluded articles describing PPIE activities without a clear link to a defined research study. For example, we excluded articles on research question priority setting with PPIE participants when it was unclear whether the identified research priorities informed a subsequent research study [26, 27], as well as articles conducting PPIE during tool or intervention development that did not refer to a subsequent study testing its application [28].

### 2.4 Study Screening

The Rayyan platform [29] was used to identify duplicates and to carry out title and abstract screening. One author (VS) screened abstracts and titles against inclusion and exclusion criteria, and any uncertainties were discussed with a second author (CH). The full text screening was completed by one author (VS), and a second author was consulted in case of uncertainties in the decision‐making process (CH).

### 2.5 Data Extraction and Synthesis

Data extracted from each original research article included year of publication, journal, country, study design, area of research, PPIE definition, number and type of PPIE participants, PPIE participant training, age of minor PPIE participants, recruitment methods, remuneration, timepoint of PPIE, level of involvement, PPIE evaluation, benefits of PPIE, positive impact of PPIE, barriers of PPIE, negative impacts of PPIE, and PPIE toolkits. Data extraction was conducted by one author (JS) and verified by a second (VS). Discrepancies were resolved through consensus following discussion.

### 2.6 Data Analysis

Given the substantial variation in PPIE activities across the included original research articles and the inconsistent reporting quality, a narrative summary and thematic synthesis was the most suitable approach to present the results. Therefore, no meta‐analysis was used to combine the individual elements of PPIE of each included original research article. If the level of involvement in a study was not described using a model or reference, we estimated the level of involvement based on the wording or the context itself. The completeness of reporting of important information was assessed using the GRIPP2 short form checklist [9]. The quality of PPIE in the included original research articles was assessed using the critical appraisal criteria developed by Wright et al. [1].

## 3 Results

### 3.1 Study Search and Selection Results

**Figure 1:**
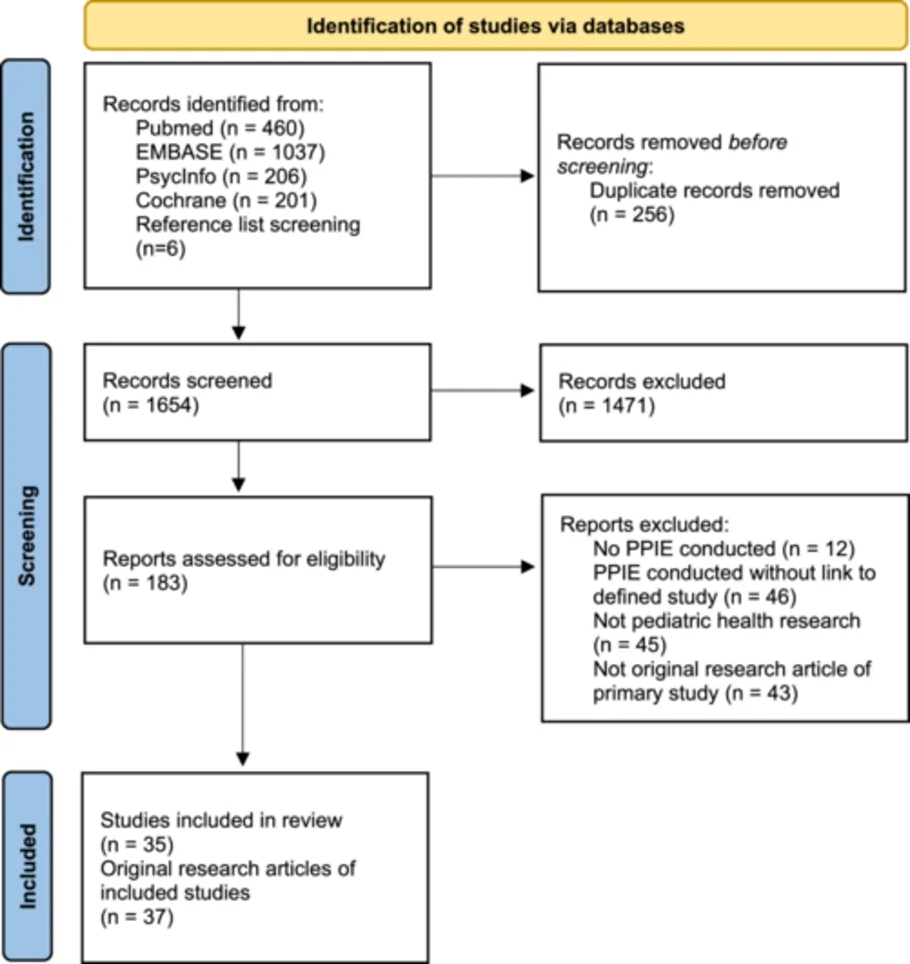
PRISMA flow diagram for publication search and selection process for the systematic review on PPIE in pediatric health research. The systematic review included 37 original research articles of 35 studies.

The search and selection process is illustrated in Figure 1. Systematic search of the electronic databases and screening of reference lists of included publications identified 1910 possibly relevant references. A total of 37 original research articles met the eligibility criteria. In two instances, the same study was described in two separate original research articles, resulting in 35 distinct studies being included in this review.

Key characteristics of the included original research articles are presented in Table 1. Most papers (*n* = 28; 76%) integrated PPIE throughout the manuscript [30, 31, 32, 33, 34, 35, 36, 37, 38, 39, 40, 41, 42, 43, 44, 45, 46, 47, 48, 49, 50, 51, 52, 53, 54, 55, 56, 57], while some (*n* = 9; 24%) addressed it only in a dedicated section [58, 59, 60, 61, 62, 63, 64, 65, 66]. The reporting of PPIE in the original research articles was variable and often incomplete, as reflected in the reporting quality assessment using the GRIPP2 short form checklist (Appendix 3). Consequently, the critical appraisal indicated that the quality of the reported PPIE activities was most often low (Appendix 4). Studies were conducted in Europe (*n* = 20; 57%) [31, 33, 34, 35, 40, 41, 42, 45, 47, 49, 50, 52, 54, 55, 56, 59, 61, 62, 63, 64, 66], North America (*n* = 14; 40%) [30, 32, 36, 38, 39, 43, 44, 46, 48, 51, 53, 57, 58, 60, 65] and Africa (*n* = 1; 3%) [37]. All included studies were published between 2010 and 2024, most of them after 2015 (*n* = 28; 80%) [30, 31, 32, 33, 35, 36, 39, 40, 41, 42, 44, 45, 46, 47, 48, 49, 51, 52, 53, 54, 55, 56, 59, 60, 61, 62, 63, 64, 65, 66]. Among the 35 included studies, 18 were randomized controlled trials (51%) [30, 31, 32, 34, 35, 38, 40, 42, 43, 46, 47, 48, 49, 55, 56, 58, 62, 64, 65, 66], nine were qualitative research studies (26%) [33, 39, 44, 45, 50, 52, 54, 59, 60], seven were observational studies (20%) [36, 37, 51, 53, 57, 61, 63], and one was a not randomized intervention study (3%) [41]. We included six pilot and feasibility studies [36, 47, 49, 58, 62, 66]. The studies focused on the following health research domains: autoimmune diseases and chronic conditions (*n* = 6; 17%) [43, 44, 49, 50, 59, 63], neurology (*n* = 4; 11%) [34, 35, 41, 61], mental health (*n* = 3; 9%) [33, 40, 52, 56], asthma (*n* = 3; 9%) [30, 31, 48], neonatology (*n* = 3; 9%) [42, 47, 64], metabolic health (*n* = 3; 9%) [39, 46, 51, 65], emergency, intensive and palliative care (*n* = 3; 9%) [45, 53, 55], sexual and substance related health behaviors (*n* = 3; 9%) [38, 58, 66], environmental health (*n* = 2; 6%) [36, 57], research participation (*n* = 1; 3%) [54], child welfare (*n* = 1; 3%) [37], gut health (*n* = 1; 3%) [62], oncology (*n* = 1; 3%) [60], and dental health (*n* = 1; 3%) [32].

**Table 1:** Characteristics of included original research articles in the systematic review on PPIE in pediatric health research.

Authors	Year	Country	Study Design	Research area	PPIE participant type	PPIE participant recruitment	PPIE method(s)	Timepoint of engagement
Allen et al. [58]	2013	USA	RCT, pilot	Sexual and substance related health behaviors	Parents	Not available	Not available	4
Antal et al. [30]	2017	USA	RCT	Asthma	Parents, adolescents	Not available	Focus group	6
Boote et al. [31]	2016	UK	RCT	Asthma	Child patients, parents of patients	Respiratory clinic, letter	Focus group	3, 4
Chi et al. [32]	2018	USA	RCT	Dental Health	Caregiver	Dental hygiene program	One on one interview by telephone	4
Coyne et al. [59]	2024	Ireland	Qualitative research study	Autoimmune disease and chronic condition	Adolescent patients, parents of patients	Not available	Not available	4, 6
Dennehy et al. [33]	2019	Ireland	Qualitative research study	Mental Health	Adolescents	School	In‐person research sessions	4, 5, 7, 8, 9
Edwards et al. [34]	2011	UK	RCT	Neurology	Parents of patients	Charity	Interview	4
Flegg et al. [60]	2020	Canada	Participatory priority setting	Oncology	Parents, Patient representatives	Not available	Video conference, one on one meeting	4, 6, 7, 10, 11
Gignac et al. [61]	2021	Spain	Cross sectional study	Neurology	Adolescents	School	School class	4
Hall et al. [35]	2019	UK	RCT	Neurology	Parents	Study team contacts, parent support group	Workshop	4, 10
Hall et al. [62]	2021	UK	RCT, pilot	Gut health	Children and young people (some are patients), parents of patients	Study team contacts, preexisting young person advice groups	One on one meeting, whole group meeting (personally and virtually)	1, 2, 4, 5, 6, 11
Haynes et al. [36]	2016	USA	Cross sectional study, pilot	Environmental health	Faculty and students, community residents, parents	Not available	Meetings, conference calls	4, 5, 10, 11
Kamanda et al. [37]	2013	Kenya	Cohort study	Child welfare	Caregiver, community health workers, community members and their key leaders, patient representatives	Applications, key leaders helped to find community members	Community assembly	1, 4, 5, 6, 7, 9, 11
Kaufman et al. [38]	2014	USA	Group randomized trial	Sexual and substance related health behaviors	Young people, community advisory board	Through community tribal members	Calls, in‐person meetings, in‐person workshops	4, 5
Kearsley‐Fleet et al. [63]	2024	UK	Cohort study	Autoimmune disease and chronic condition	Patient representatives, parents of patients	Not available	Not available	1, 4, 10
Kendal et al. [52]	2017	UK	Qualitative research study	Mental health	Adolescents	Study team contacts, flyers, e‐mails, college	Focus group	4, 5, 6, 7, 10
Kim et al. [39]	2020	USA	Qualitative research study	Metabolic health	Adolescents	Open application, interviews	Small group meeting, Full group meeting	1, 4, 5, 7, 8, 11
Knisley et al. [53]	2021	Canada	Cross sectional study	Emergency, intensive and palliative care	Caregiver	Preexisting parent/public advisory groups of partner organizations	Survey	5
Lampa et al. [40]	2022	Sweden	RCT	Mental health	Parents	Public language school	Face to face meeting, online meeting	2, 4, 6, 8, 11
Langer et al. [41]	2022	Germany	Intervention study	Neurology	Parents, representatives of patient organizations	E‐Mail to all affected families in city	Workshop	4, 6
Levene et al. [42]	2022	UK	RCT	Neonatology	Parents, charity collaborators	Charity	Questionnaire, video conference	1, 2, 4, 5, 6, 11
Lowes et al. [43]	2011	USA	RCT	Autoimmune disease and chronic condition	Adolescents and young people patients, parents of patients	Support groups	Meeting	4
Luchtenberg et al. [54]	2020	Netherlands	Qualitative research study	Research participation	Children (some are patients)	Patient support organizations, primary schools, hospitals, social media, word of mouth	One on one meetings at home, group meetings	7
March et al. [44]	2021	USA	Qualitative research study	Autoimmune disease and chronic condition	Parents of patients	Study team contacts	Virtual meeting, Individual phone calls	4, 5, 7, 9
Mitchell et al. [45]	2021	UK	Qualitative research study	Emergency, intensive and palliative care	Young people (some are patients)	Preexisting young people's advisory groups	Meeting	1, 4, 6, 9, 10, 11
Monk et al. [64]	2019	UK	RCT	Neonatology	Parents	Charity	Workshop	2, 3, 4, 5, 6, 10, 11
Oridota et al. [46]	2023	USA	RCT	Metabolic health	Young people	Previous participation in pendant study, school	Not available	Not available
Rayment et al. [47]	2017	UK	RCT, pilot	Neonatology	Mothers	Children's centers, church, preexisting advisory group	Discussion group	4, 6
Shelef et al. [48]	2016	USA	RCT	Asthma	Parents	Not available	Face to face meeting, telephone call	4, 5
Sherratt et al. [49]	2018	UK	RCT, pilot	Autoimmune disease and chronic condition	Parents, children patients	Clinic	Interview	4, 5
Tume et al. [55]	2016	UK	Cluster RCT	Emergency, intensive and palliative care	Parents of patients, patient representative, young people	Posters and leaflets in parent's room and conference, charity, network	Interview (face to face, telephone), focus group	4
van Staa et al. [50]	2010	Netherlands	Qualitative research study	Autoimmune disease and chronic condition	Adolescents, young people	Leaflets in clinic, website	Meeting	4, 6, 11
Vangeepuram et al. [65]	2017	USA	RCT	Metabolic health	Not available	Not available	Not available	4, 5, 6, 7, 8, 11
Walton et al. [51]	2018	Canada	Cohort study	Metabolic health	Parents	Pilot study participants	Face to face meeting, e‐mail	2, 4, 5, 6, 9, 10, 11
Warner et al. [56]	2021	Sweden	RCT	Mental health	Parents, young person	Local education centers	Meeting	4, 5
Warren et al. [57]	2014	USA	Observational study	Environmental health	Women (mothers)	Word‐of‐mouth, community boards, community meetings	Meeting	5
Watson et al. [66]	2017	UK	RCT, pilot	Sexual and substance related health behaviors	Young people patients, parents	Charity, clinic	Face to face meeting, post card, text message, e‐mail, telephone, focus group	4, 5, 6, 7, 10, 11

*Note:* Patients and the public were engaged at 11 different timepoints (1: research question, 2: funding application, 3: ethics application, 4: study design, 5: recruitment, 6: study process, 7: data analysis, 8: study results, 9: study evaluation, 10: paper writing, 11: dissemination).

### 3.2 PPIE Participants Characteristics

**Figure 2:**
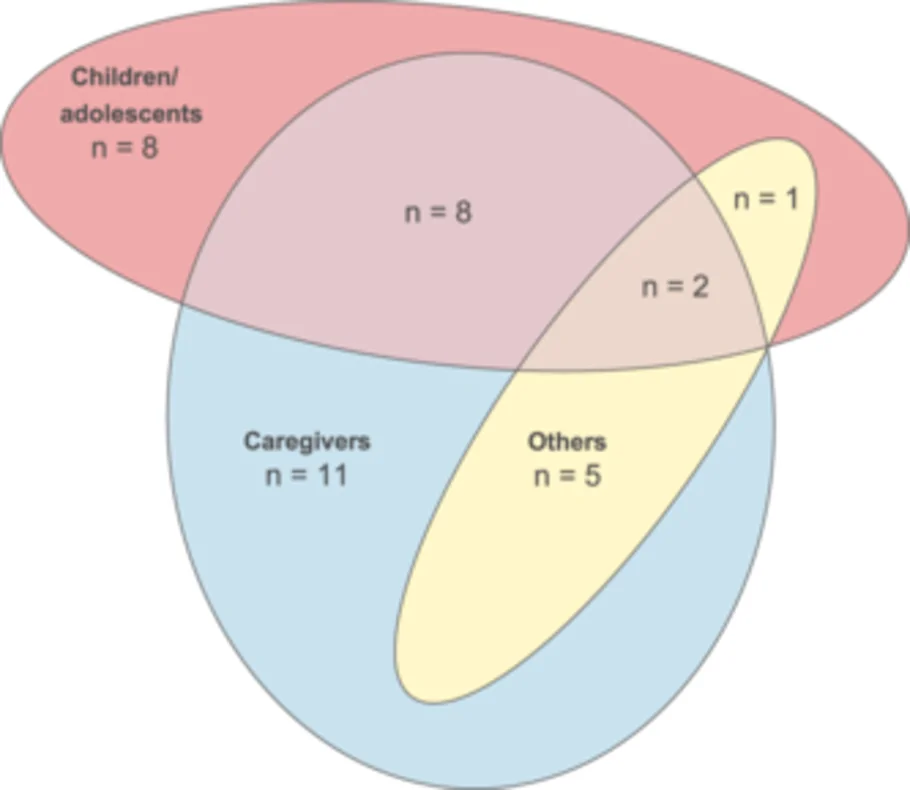
Venn diagram showing the overlap of studies that involved children/adolescents, caregivers or others as PPIE participants in pediatric health research. Shown are studies that involved children/adolescents (*n* = 19; 54%), caregivers (*n* = 26; 74%) or others (*n* = 8; 23%). Others include adult patient representatives (*n* = 3; 9%), community members (*n* = 3; 9%) and representatives from patient organizations or charities (*n* = 2; 6%).

PPIE participants included minors, parents or caregivers, or a combination of these groups in most studies, as shown in Figure 2. In total, 19 studies (54%) described the involvement of children, adolescents or young people as PPIE participants [30, 31, 33, 37, 38, 39, 43, 45, 46, 49, 50, 52, 54, 55, 56, 59, 61, 62, 65, 66], and 26 studies (74%) described the involvement of parents or caregivers as PPIE participants [30, 31, 32, 34, 35, 36, 37, 40, 41, 42, 43, 44, 47, 48, 49, 51, 53, 55, 56, 57, 58, 59, 60, 62, 63, 64, 66]. PPIE participants were exclusively children, adolescents or young people in eight studies (23%) [33, 39, 45, 46, 50, 52, 54, 61, 65], exclusively parents or caregivers in 10 studies (29%) [32, 34, 35, 44, 47, 48, 51, 53, 58, 64], or a combination of both groups in eight studies (23%) [30, 31, 40, 43, 49, 56, 59, 62, 66]. The age of children, adolescents or young people involved as PPIE participants was 7–25 years [30, 31, 33, 39, 45, 46, 49, 50, 52, 54, 61, 62, 66]. Only a few studies included children up to the age of 12 years (*n* = 6; 17%) [30, 31, 45, 49, 54, 62]. In some studies, also adult patient representatives (*n* = 3; 9%) [55, 60, 63], community members (*n* = 3; 9%) [37, 38, 57], or representatives from patient organizations or charities (*n* = 2; 6%) [41, 42] were involved. All studies reported that all or a subset of PPIE participants had experience with the condition under study, either as patients themselves or as a caregiver [30, 31, 32, 33, 34, 35, 36, 37, 38, 39, 40, 41, 42, 43, 44, 45, 46, 47, 48, 49, 50, 51, 52, 53, 54, 55, 56, 57, 58, 59, 60, 61, 62, 63, 64, 65, 66]. Most often, PPIE participants were recruited via clinical sites (*n* = 6; 17%) [31, 49, 50, 54, 55, 66], study team contacts or previous study participation (*n* = 6; 17%) [35, 44, 46, 51, 52, 62], charities (*n* = 5; 14%) [34, 42, 55, 64, 66], schools (*n* = 5; 14%) [33, 40, 46, 54, 61], existing young person advisory groups (*n* = 4; 11%) [45, 47, 53, 62], or support groups (*n* = 3; 9%) [35, 43, 54]. In some studies, PPIE participants received training about the study or about research in general (*n* = 17; 49%) [31, 33, 34, 38, 39, 43, 45, 46, 47, 48, 49, 50, 54, 57, 61, 62, 64]. It was mentioned that the training was not extensive in order to ensure the validity of the lay views [43, 54]. As a reimbursement, PPIE participants received vouchers (*n* = 6; 17%) [31, 43, 51, 54, 62, 66], direct payments (*n* = 5; 14%) [40, 42, 44, 50, 56, 66], refund of travel expenses (*n* = 5; 14%) [31, 43, 60, 62, 66], a certificate of attendance (*n* = 3; 9%) [31, 33, 54], or free childcare (*n* = 2; 6%) [51, 62]. There was no information on PPIE participant reimbursement in 20 studies (57%) [30, 32, 34, 35, 36, 39, 41, 45, 46, 47, 49, 52, 53, 55, 57, 58, 59, 61, 63, 64, 65].

### 3.3 Timepoint of PPIE Activities

**Figure 3:**
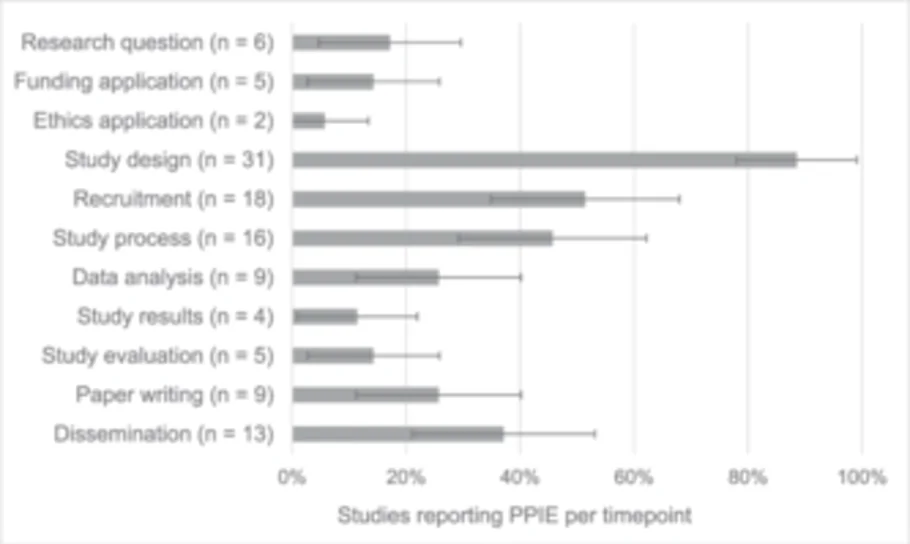
Bar chart showing the percentage of included studies that reported PPIE activities in different study timepoints (*n* = 35) in pediatric health research.

PPIE participants were involved in eleven different stages of studies, shown in Figure 3. Some studies reported PPIE participants being involved in only a single stage (*n* = 10; 29%) [30, 32, 34, 43, 53, 54, 55, 57, 58, 61]. Most studies described PPIE activities across multiple stages (*n* = 25; 71%) [31, 33, 35, 36, 38, 39, 40, 41, 42, 44, 45, 47, 48, 49, 50, 52, 56, 59, 60, 62, 63, 65, 66] and a few studies reported PPIE in up to seven distinct stages (*n* = 3; 9%) [37, 51, 64]. PPIE was conducted in the study design phase in 89% of studies (*n* = 31) [31, 32, 33, 34, 35, 36, 37, 38, 39, 40, 41, 42, 43, 44, 45, 47, 48, 49, 50, 51, 52, 55, 56, 58, 59, 60, 61, 62, 63, 64, 65, 66], while only 11% of studies (*n* = 4) did not report PPIE in this stage [30, 53, 54, 57]. For example, PPIE participants were involved in the definition of inclusion and exclusion criteria [33], the estimation of effort and burden of study participation, and in the determination of relevant study outcomes [32]. PPIE participants were less involved in the early steps of studies. Only 17% of studies (*n* = 6) involved PPIE participants during research question definition [37, 39, 42, 45, 62, 63].

### 3.4 PPIE Methods

Studies reported a variety of methods used to conduct PPIE activities. In many studies (*n* = 20; 57%), PPIE participants and researchers interacted in a meeting setting. Meetings were organized either in‐person or virtually, and as one‐to‐one or whole group meetings [33, 36, 37, 38, 39, 40, 42, 43, 44, 45, 47, 48, 50, 51, 54, 56, 57, 60, 62, 66]. In some studies, group meetings were specified as focus groups (*n* = 5; 14%) [30, 31, 52, 55, 66], or workshops (*n* = 4; 11%) [35, 38, 41, 64]. In some cases, PPIE participants were offered one‐to‐one contact after they were unable to attend whole group meetings [44, 60, 62]. In addition, researchers involved PPIE participants through interviews remote or face to face (*n* = 4; 11%) [32, 34, 49, 55].

### 3.5 Level of Involvement

Few authors classified the level of involvement by providing a reference model. Knisley et al. [53] rated the level of involvement in their publication as consultation based on the IAP2 Spectrum of Public Participation [67]. Langer et al. [41] referred to the spectrum of engagement described by Ball et al. [68], and classified the involvement in their study as consultation. The level of involvement used by Lowes et al. [43] was labeled as collaboration, based on the categorizations by Kirby et al. [69]. Watson et al. [66] referred to the definitions for levels of involvement described by Hanley et al. [70]. They started with consultation, but exceeded to a more collaborative level, depending on the expressed interest of PPIE participants. Also, the level of involvement in the study by Walton et al. [51] ranged from information and consultation up to parent control. They visualized the different levels in their ladder of parent participation. However, the level of involvement was not described using a model or reference in most studies (*n* = 30, 86%). Among those, in few studies (*n* = 10; 29%), involvement was fully or partly high level, approaching co‐production [33, 35, 37, 39, 46, 50, 52, 57, 60, 62, 65], whereas in most studies (*n* = 20; 57%), the level of involvement was rather low, tending towards consultation [30, 31, 32, 34, 36, 38, 40, 42, 44, 45, 47, 48, 49, 54, 55, 56, 58, 59, 61, 63, 64].

### 3.6 Benefits and Positive Impacts of PPIE

Benefits and positive impacts, as well as barriers and negative impacts of PPIE are summarized in Table 2. Some studies reported a positive effect of PPIE on recruitment or retention rates [34, 39, 51, 58]. For instance, Walton et al. [51] conducted a longitudinal cohort study to identify early life risk factors for chronic disease and obesity and to test the effectiveness of a family‐based intervention aimed at supporting healthy behaviors in early childhood. They attributed their effective recruitment approach and high retention rate to the contributions of PPIE participants. Some studies explicitly mentioned that PPIE participants highlighted aspects that had not been considered by the researchers [35, 47, 61] and described PPIE as a key contributor to the success of the trial [34, 37, 62, 64]. Several studies described a personal benefit for PPIE participants. Involvement in research was perceived as a learning experience about research [33, 39, 40, 45, 46, 50, 54, 57, 61, 62, 66] and was associated with a sense of accomplishment derived from stepping out of one's comfort zone [33, 39]. Participation in PPIE activities resulted in a gain of confidence [50, 51, 54, 57, 62]. PPIE participants valued the opportunity to connect and share experiences with people navigating similar challenges [39, 40, 43, 46, 50, 51, 66] and gained knowledge for their own health from the research topic of the study [33, 46, 54].

**Table 2:** Benefits, positive impacts, barriers and negative impacts of PPIE on pediatric health research and PPIE participants.

	Benefits and positive impact	Barriers and negative impact
Research	Increased recruitment and retention rates [34, 39, 51, 58]	Financial and time resources [31, 33, 35, 37, 38, 42, 50, 54, 57, 60, 61]
	Awareness of points not on researchers minds [35, 47, 61]	Challenging recruitment of representative PPIE participants [31, 34, 40, 42, 46, 47, 50, 51, 52, 54, 55, 58, 62, 66]
	Stronger relevance of study findings [37]	Sustaining engagement and maintaining contact to PPIE participants [35, 50, 62, 66]
	Lower study participant burden through trial design [42]	Aligning PPIE input with overall study aim [35, 37, 48]
PPIE Participants	Learning about research [33, 39, 40, 45, 46, 50, 54, 57, 61, 62, 66]	Intimidation by researchers and power imbalance between adults and children [33, 40, 43, 46]
	Step out of comfort zone [33, 39]	Fear of negative consequences after sharing experiences on a sensitive topic [66]
	Gain in confidence [50, 51, 54, 57, 62]	Frame for PPIE impact created by researchers [41]
	Gain in knowledge for own health [33, 46, 54]	
	Building connections and sharing experiences with people in similar life situations [39, 40, 43, 46, 50, 51, 66]	

### 3.7 Barriers and Negative Impacts of PPIE

A recurring reported barrier was the need for additional financial resources to support PPIE activities [31, 35, 38, 42, 50, 57, 60]. In addition, PPIE activities were time consuming for researchers and PPIE participants [31, 33, 37, 38, 50, 54, 61]. Several studies highlighted the challenging recruitment of PPIE participants [31, 47, 51, 52, 54, 55, 66] and the difficulty of achieving representativeness or diversity among PPIE participants [34, 40, 42, 46, 50, 51, 58, 62, 66]. Few studies described the challenges of sustaining PPIE participants' involvement and maintaining ongoing contact [35, 50, 62, 66]. For instance, van Staa et al. [50] involved nine adolescents as PPIE participants in a qualitative research study about the needs of chronically ill adolescents in the hospital setting. Chronic illness among PPIE participants presented barriers to maintaining consistent levels of participation. The absence of anonymity or the presence of researchers or may have created an intimidating environment for PPIE participants, particularly for children. This may have constrained the scope and authenticity of their input and feedback [33, 40, 43, 46]. In addition, aligning PPIE participants input with researchers' study objectives sometimes presented a challenge [35, 37, 48]. Watson et al. [66] involved 17 young people in a randomized controlled feasibility trial testing an intervention for young people who misuse alcohol and drugs. In this study, some PPIE participants with personal experience withheld disclosure of their involvement due to fear of stigma or negative consequences for their career or personal future, highlighting a possible risk associated with PPIE.

## 4 Discussion

### 4.1 Summary

This systematic review highlights that PPIE is valuable in pediatric health research. In most studies, PPIE participants were children, adolescents or caregivers, with caregivers taking part more often than the young people themselves. This indicates that tailored approaches for the involvement of children and adolescents are needed. PPIE participants were often recruited via charities, patient organizations, existing support or advisory groups, clinical sites, or study team contacts. By this approach, only a certain population might be reached, which reinforces the challenge of low representativeness of PPIE participants among patients and the public. Therefore, more open and inclusive approaches to PPIE participant recruitment should be considered, such as local newsletters or general practitioner practices. PPIE participants were mainly involved in study design, recruitment or in the study process, but less involved in the early steps of studies, such as definition of research question, funding application or ethics application. Researchers should be encouraged to involve PPIE participants in the beginning of their study to increase the clinical relevance of the research, and to examine whether early involvement can help move involvement towards a co‐design level. PPIE activities mainly included meetings, focus groups, workshops, and interviews. To enhance the benefit of PPIE, the use of novel methods could be considered, such as photovoice [71]. The level of involvement varied highly between studies, from consultation to co‐design, with consultation being the predominant level of involvement. Reported positive impacts of PPIE on research included higher recruitment or retention rates, identification of issues researchers have not considered, or personal benefits for PPIE participants. In contrast, barriers to PPIE were financial costs, time investment, challenging recruitment of PPIE participants and achieving representativeness among them. A change in research culture is needed to facilitate the allocation of funding and time resources to PPIE. Our findings imply that PPIE is integrated in pediatric health research in very different ways, and each approach is connected to benefits and challenges.

### 4.2 Summary in the Context of Literature

The PPIE reporting quality of the studies in our systematic review varied greatly. This aligns with findings of Preston et al. [23]. They identified pediatric research reports and found substantial variation in the reporting of PPIE involving children and young people. Thereby, only a subset of their included papers reported PPIE according to the GRIPP2 short form checklist criteria. Assessing the quality of PPIE activities through critical appraisal was challenging because most studies provided only limited details about the activities. Bailey et al. [21] reported similar challenges in their systematic review about involving disabled children and young people as partners in research. Our findings regarding benefits and challenges are in line with the conclusions of Vanderhout et al. [3] in their scoping review about the impact of PPIE in child health research. Shen et al. [72] also concluded similar benefits and challenges of PPIE in their scoping review about the involvement of parents as co‐researchers in health research. Additionally, they suggested that the involvement of parents as co‐researchers contributed to wider dissemination. Concerning challenges of PPIE, the authors highlighted that power largely remained with the researchers, potentially leading to tokenistic participation from parents. Wyatt et al. [16] described in their umbrella review about involvement of children and young people in health research that PPIE participants were most common involved in study design, study process or study dissemination. This is in alignment with our results. In addition, they reported benefits and barriers of PPIE similar to our findings. Ennis et al. [73] found a positive association between the level of involvement and the achievement of recruitment goals in adult mental health research. Also, Crocker et al. [74] concluded from their meta‐analysis that PPIE can enhance recruitment of study participants in adult clinical trials, particularly when involving individuals with lived experience of the condition being studied. In our systematic review, a subset of studies concluded that PPIE had a beneficial effect on recruitment and retention rates. However, there was no study that included a control setting without PPIE.

Existing literature has already summarized benefits and challenges of PPIE in adult health research. Domecq et al. [13] included 142 studies about patient involvement in health research. They concluded that most studies reported benefits resulting from PPIE, such as higher recruitment and retention rates, as well as more meaningful dissemination. In addition, they emphasized that a common challenge of PPIE was to ensure non‐tokenistic practices. Mathieson et al. [12] conducted a scoping review about PPIE in implementation research. They reported barriers, as well as an impact of PPIE on research and on PPIE participants similar to the results of our systematic review. These findings show that there are parallels in benefits and challenges of PPIE between adult health research and pediatric health research, indicating that the two disciplines can learn from each other's experiences. PPIE in pediatric research differs from adult research in the way that children should be involved as PPIE participants. Some researchers may be reluctant to involve children as PPIE participants, which can partially be addressed by adhering to guidance and best practice examples [6].

In general, each participant group involved in PPIE, including children, adolescents, young people, caregivers, and adult patients, requires distinct approaches. For example, age‐appropriate involvement methods, information and communication strategies may be needed to facilitate meaningful involvement [8]. In addition, their contributions to PPIE activities may differ, as each group and each individual has distinct lived experiences, perspectives, and research priorities. Similar considerations apply to the involvement of equity‐seeking groups, such as individuals with disabilities or those from socially disadvantaged backgrounds. A more detailed synthesis of these subgroups was not possible in our review because many included original research articles did not provide sufficient information on participant characteristics or report PPIE experiences separately for different participant groups. Existing reviews focusing on specific PPIE participant groups have highlighted considerations relevant to these populations. Bailey et al. [21], in their systematic review of involving disabled children and young people as partners in research, highlighted the need for more flexible involvement approaches, particularly appropriate communication methods. Bailey et al. [14], in their review of benefits, barriers and recommendations for youth engagement in health research, emphasized the importance of considering the dynamics of power‐sharing between young people and researcher. Future research should further investigate differences in PPIE experiences across diverse PPIE participant groups, identify barriers and facilitators to conducting PPIE with several PPIE participant groups in parallel, and examine the specific considerations required for meaningfully involving children, adolescents, young people, caregivers and equity‐seeking groups in PPIE.

### 4.3 Limitations and Strengths

As a strength, this systematic review provides a comprehensive mapping of PPIE practices, methodological approaches and information on positive effects and challenges of PPIE in pediatric health research. Studies included in this systematic review were not restricted to specific disciplines in pediatric health research. Therefore, a broad range of researchers can transfer our findings to their research projects. By excluding articles that describe PPIE activities without a clear link to a defined research study, we aim to review meaningful PPIE implementation in pediatric health research. Overall, our findings agree with previous systematic reviews on similar topics [15, 20, 21, 22]. Preregistration of protocol and search strategy ensure transparency and reproducibility of this systematic review.

This systematic review has a few limitations. Study screening was conducted by a single researcher, which may have increased the risk of selection bias. We applied predefined eligibility criteria and discussed uncertainties with a second researcher. Nevertheless, independent screening by multiple researchers could have strengthened the reliability and reproducibility of study selection. We made a post‐hoc decision to exclude systematic reviews from the Results to focus on the synthesis of original research articles. Although this represents a deviation from the protocol, the systematic reviews were screened for additional eligible original research articles and remain cited in the Introduction and Discussion of this review. We may have missed some relevant studies that do not mention the conduct of PPIE in their title or abstract. Nevertheless, the GRIPP2 long form reporting checklist [9] recommends to report aim, methods, results and conclusion, as well as PPIE related keywords in the abstract of the paper. In addition, our search strategy might have overlooked some studies due to the wide variation in terminology referring to PPIE. We mitigated this by including studies using terms such as community based participatory research [58], patient centered outcomes research [32], citizen science [61] or stakeholder engagement [44] to describe PPIE. We only included paper published in English or German. Also, we could not assess the extent to which publication bias and reporting bias may have influenced the results of our systematic review. Our findings might not provide a comprehensive reflection on the levels of involvement used in pediatric health research, since information on the level of involvement was available for only part of the studies reviewed. Wyatt et al. [16] also described this limitation in their umbrella review about the involvement of children and young people in the conduct of health research. Because the reporting quality varied across studies and often did not adhere to guidelines, the frequency with which specific benefits, positive impacts, barriers or negative impacts were mentioned in studies may not reflect their true occurrence.

### 4.4 Implications for Research and Clinical Practice

This systematic review aimed to capture the benefits and barriers of PPIE activities in pediatric health research. By displaying different approaches of PPIE used in various studies, we would like to facilitate that researchers can transfer these strategies to their own research projects. This would ensure that future research is better aligned with the needs of those who directly benefit from its findings in clinical practice. It is known that discontinuation of studies due to difficulties in enrolling study participants is common in pediatric research [75]. Since PPIE can have a positive effect on recruitment or retention rates, it might thereby serve as approach to reduce research waste.

## 5 Conclusion

PPIE practices can improve the acceptability and relevance of pediatric health research. The literature describes various approaches to conduct PPIE in pediatric health research, aligned with several benefits and challenges. This indicates that PPIE requires tailored activities rather than a one size fits all approach, depending on the requirements of the research discipline, age of patients, and study design. However, a high level of involvement should be aimed for to enhance the benefits of PPIE. It is also particularly important to adhere to evidence‐informed guidelines for conducting and reporting PPIE that have been established in recent years. This will facilitate mutual learning and support the implementation of meaningful PPIE in pediatric health research studies.

## Author Contributions

**Veronika Stadler:** conceptualization, methodology, data curation, formal analysis, supervision, validation, investigation, visualization, project administration, writing – original draft, writing – review and editing. **Rebeca Mozun:** conceptualization, methodology, supervision, validation, writing – review and editing. **Jannik Strohm:** data curation, investigation, writing – review and editing. **SwissPedHealth consortium:** funding acquisition. **Chloé Caruso:** conceptualization, writing – review and editing, methodology. **Klara M. Posfay‐Barbe:** conceptualization, methodology, supervision, writing – review and editing, project administration. **Cornelia Hagmann:** conceptualization, methodology, project administration, writing – review and editing, validation, supervision.

## Conflicts of Interest

The authors declare no conflicts of interest.

## Clinical Trial Registration

This systematic review was registered with OSF (10.17605/OSF. IO/WPFUM) and PROSPERO (CRD420251035067).

## Take Home Message

PPIE is beneficial for pediatric health research and its positive impact can be enhanced by adherence to conduction and reporting guidelines and by aiming for a high level of involvement.

## Collaborators

SwissPedHealth consortium: Andrea Agostini, Anita Rauch, Audrey van Drogen, Aurélie Martin Necker, Ben D Spycher, Christian Kahlert, Christopher B Forrest, Claudia E Kuehni, Cornelia Hagman, D Sean Froese, Daphné Chopard, Dylan Lawless, Effy Vayena, Eirini I Petrou, Emanuele Palumbo, Eric Giannoni, Fabiën N Belle, Ioannis Xenarios, Jacques Fellay, Jana Pachlopnik Schmid, Julia A Bielicki, Julia E Vogt, Kathrin Hofmann, Katrin Männik, Keith Harshman, Kelly Ormond, Klara Posfay‐Barbe, Léa Ho Dac, Lorenz M Leuenberger, Luregn J Schlapbach, Manon Jaboyedoff, Mariam Ait Oumelloul, Martin Stocker, Matthias R Baumgartner, Nicola Zamboni, Nicole Goebel, Patrick G A Pedrioli, Philipp Latzin, Rebeca Mozun, Roger Lauener, Sandra Goetze, Seraina Prader, Simon Boutry, Sven Schulzke, Tatjana Welzel, Thomas M Sutter, Varvara Dimopoulou, Vito Rt Zanotelli, Xeni Deligianni, Xenia Bovermann, Yara Shoman, Anna Hartung.

## Supporting information


**Appendix 1:** Lay summary of systematic review on PPIE in pediatric health research.

**Appendix 2:** Search strategies for databases in the systematic review on PPIE in pediatric health research.

**Appendix 3:** PPIE reporting quality assessment using GRIPP2 short form checklist for studies included in the systematic review on PPIE in pediatric health research.

**Appendix 4:** Critical appraisal criteria according to Wright et al. [ 1] used to assess the quality of PPIE in the original research articles included in the systematic review on PPIE in pediatric health research. Assigned categories are based on the Critical Appraisal Skills Programme (CASP) Qualitative Studies Checklist [ 2]. “Can't tell” indicates that the appraisal criterion cannot be assessed because the original research article provides insufficient or no relevant information.

## Data Availability

The data supporting the findings of this systematic review are available in the appendix.
