# Supplementary material for: Patient and Public Involvement and Engagement in Pediatric Health Research: A Systematic Review
Source: Health Expect. 2026 Sep 4;29(5):e70862. doi: 10.1111/hex.70862 (PMC13543670; doi:10.1111/hex.70862)
Supplement: Supplementary file 1 [file HEX-29-e70862-s001.docx]

Appendix 4: Critical appraisal criteria according to Wright et al. [1] used to assess the quality of PPIE in the original research articles included in the systematic review on PPIE in pediatric health research. Assigned categories (Yes, Can’t tell) are based on the Critical Appraisal Skills Programme (CASP) Qualitative Studies Checklist [2].

| Research activity | Appraisal criteria |
| --- | --- |
| Planning and project design | 1. Is the rationale for involving users clearly demonstrated?  *Consider the following: (a) Have the researchers explained the rationale for user involvement?*    2. Is the level of user involvement appropriate?  *Consider the following: (a) Have the researchers explained and justified the level of user involvement? (e.g. have they discussed whether the study involves user consultation, user collaboration or user control?) b) Have the researchers discussed the nature of tasks users were asked to perform (e.g. identifying the research question, selecting the research method, commenting on information sheets, data collection, data analysis, dissemination?)* |
| Recruitment and training | 3. Is the recruitment strategy appropriate?  *Consider the following: (a) Have the researchers explained how users have been identified? (b) Have attempts been made to involve a wide cross‐section of interests where appropriate (e.g. ethnic minorities, age, gender)? (c) Have the researchers discussed the ‘credentials’ of the users involved? (E.g. Do the researchers discuss why the users involved are appropriate to meeting the aims of the involvement activity?)*  4. Is the nature of training appropriate?  *Consider the following: (a) Have the researchers discussed the nature of the training provided? (b) Is the nature and extent of the training justified by the researchers? (e.g. Do the researchers discuss how the training meets the needs of the users during the course of the study?) (c) Has an account been given of user involvement training for professional researchers, where necessary?* |
| Data collection and analysis | 5. Has sufficient attention been given to the ethical considerations of user involvement and how these were managed?  *Consider the following: (a) Do the researchers discuss ethical issues relating to the involvement of users in research (e.g. fatigue, the emotional demands of data collection)? (b) Are there any discussions about the management of ethical issues (e.g. provision of adequate information about research tasks, peer supervision)?*    6. Has sufficient attention been given to the methodological considerations of user involvement and how these were managed?  *Consider the following: (a) Have the researchers discussed methodological issues relating to user involvement in research (e.g. potential impact on the quality of the data)? (b) Do the researchers discuss how methodological issues are managed (e.g. how differences in interpretations of qualitative data are negotiated?)* |
| Dissemination | 7. Have there been any attempts to involve users in the dissemination of findings?  *Consider the following: (a) Have users been involved in the writing of the publication / funding application? (b) Have the researchers described how the findings have been disseminated to participants and service users? (c) Are findings disseminated appropriately where necessary (e.g. translation of findings into different languages, provision of interim findings to participants in receipt of palliative care)?* |
| Evaluation and impact assessment | 8. Has the ‘added‐value’ of user involvement been clearly demonstrated?  *Consider the following: (a) Do the researchers discuss what difference involving users in the design and conduct of the research has made to the research process? (I.e. Have the researchers considered whether the study and findings would look any different if users were not involved?) (b) Do the researchers support the claims for the benefits of user involvement with examples from the research project?*  9. Have there been any attempts to evaluate the user involvement component of the research?  *Consider the following: (a) Have the researchers discussed the evaluation of the impact of user involvement on the research project (e.g. impact on the length of the study, the financial cost of involvement activities, cost‐benefit analyses)? (b) Do the researchers support claims about the impact of user involvement with examples from the evaluation?* |

| **Author, Year** | **1** | **2** | **3** | **4** | **5** | **6** | **7** | **8** | **9** |
| --- | --- | --- | --- | --- | --- | --- | --- | --- | --- |
| Allen, 2013 | Yes | Can’t tell | Can’t tell | Can’t tell | Can’t tell | Can’t tell | Can’t tell | Can’t tell | Can’t tell |
| Antal, 2017 | Yes | Yes | Can’t tell | Can’t tell | Can’t tell | Can’t tell | Can’t tell | Can’t tell | Can’t tell |
| Boote, 2016 | Yes | Yes | Can’t tell | Can’t tell | Can’t tell | Yes | Can’t tell | Yes | Can’t tell |
| Chi, 2018 | Yes | Yes | Yes | Can’t tell | Can’t tell | Can’t tell | Yes | Yes | Can’t tell |
| Coyne, 2024 | Yes | Can’t tell | Can’t tell | Can’t tell | Can’t tell | Can’t tell | Can’t tell | Can’t tell | Can’t tell |
| Dennehy, 2019 | Yes | Yes | Yes | Yes | Yes | Can’t tell | Can’t tell | Yes | Yes |
| Edwards, 2011 | Yes | Yes | Yes | Can’t tell | Can’t tell | Can’t tell | Can’t tell | Yes | Can’t tell |
| Flegg, 2020 | Yes | Yes | Can’t tell | Can’t tell | Can’t tell | Can’t tell | Yes | Can’t tell | Can’t tell |
| Gignac, 2021 | Yes | Yes | Yes | Yes | Can’t tell | Can’t tell | Can’t tell | Yes | Can’t tell |
| Hall, 2019 | Yes | Yes | Can’t tell | Can’t tell | Can’t tell | Yes | Can’t tell | Yes | Can’t tell |
| Hall, 2021 | Yes | Yes | Yes | Yes | Can’t tell | Yes | Yes | Yes | Yes |
| Haynes, 2016 | Yes | Can’t tell | Yes | Can’t tell | Can’t tell | Can’t tell | Yes | Yes | Can’t tell |
| Kamanda, 2013 | Yes | Yes | Yes | Can’t tell | Yes | Can’t tell | Yes | Yes | Can’t tell |
| Kaufman, 2014 | Yes | Yes | Yes | Yes | Can’t tell | Can’t tell | Can’t tell | Yes | Can’t tell |
| Kearsley-Fleet, 2024 | Yes | Yes | Can’t tell | Can’t tell | Can’t tell | Can’t tell | Yes | Yes | Can’t tell |
| Kendal, 2017 | Yes | Yes | Yes | Can’t tell | Can’t tell | Can’t tell | Yes | Yes | Can’t tell |
| Kim, 2020 | Yes | Yes | Yes | Yes | Can’t tell | Can’t tell | Yes | Yes | Yes |
| Knisley, 2021 | Yes | Yes | Yes | Can’t tell | Can’t tell | Can’t tell | Can’t tell | Yes | Can’t tell |
| Lampa, 2022 | Yes | Yes | Yes | Can’t tell | Yes | Yes | Yes | Yes | Yes |
| Langer, 2022 | Yes | Yes | Yes | Can’t tell | Can’t tell | Can’t tell | Can’t tell | Yes | Can’t tell |
| Levene, 2022 | Yes | Yes | Yes | Can’t tell | Can’t tell | Can’t tell | Yes | Yes | Yes |
| Lowes, 2011 | Yes | Yes | Yes | Yes | Yes | Yes | Can’t tell | Yes | Yes |
| Luchtenberg, 2020 | Yes | Yes | Yes | Yes | Yes | Yes | Can’t tell | Yes | Yes |
| March, 2021 | Yes | Yes | Yes | Can’t tell | Can’t tell | Can’t tell | Can’t tell | Yes | Yes |
| Mitchell, 2021 | Yes | Yes | Yes | Yes | Can’t tell | Can’t tell | Yes | Yes | Yes |
| Monk, 2019 | Yes | Yes | Can’t tell | Can’t tell | Can’t tell | Can’t tell | Yes | Yes | Can’t tell |
| Oridota, 2023 | Yes | Yes | Yes | Yes | Yes | Can’t tell | Yes | Yes | Yes |
| Rayment, 2017 | Yes | Yes | Yes | Can’t tell | Yes | Can’t tell | Can’t tell | Yes | Can’t tell |
| Shelef, 2016 | Yes | Yes | Yes | Can’t tell | Can’t tell | Yes | Can’t tell | Yes | Can’t tell |
| Sherratt, 2018 | Yes | Yes | Yes | Can’t tell | Can’t tell | Can’t tell | Can’t tell | Yes | Can’t tell |
| Tume, 2016 | Yes | Yes | Yes | Can’t tell | Can’t tell | Can’t tell | Can’t tell | Yes | Can’t tell |
| van Staa, 2010 | Yes | Yes | Yes | Yes | Can’t tell | Yes | Yes | Yes | Yes |
| Vangeepuram, 2017 | Yes | Yes | Can’t tell | Can’t tell | Can’t tell | Can’t tell | Can’t tell | Yes | Can’t tell |
| Walton, 2018 | Yes | Yes | Yes | Can’t tell | Can’t tell | Can’t tell | Yes | Yes | Yes |
| Warner, 2021 | Yes | Yes | Yes | Can’t tell | Yes | Yes | Can’t tell | Yes | Yes |
| Warren, 2014 | Yes | Yes | Yes | Yes | Can’t tell | Yes | Can’t tell | Yes | Yes |
| Watson, 2017 | Yes | Yes | Yes | Can’t tell | Yes | Yes | Yes | Yes | Yes |

“Can’t tell” indicates that the appraisal criterion cannot be assessed because the original research article provides insufficient or no relevant information.

1.	Wright, D., et al., *Critical appraisal guidelines for assessing the quality and impact of user involvement in research.* Health Expect, 2010. **13**(4): p. 359-68.

2.	Critical Appraisal Skills Programme. *CASP Qualitiative Studies Checklist*. 2024; Available from: <https://casp-uk.net/casp-tools-checklists/qualitative-studies-checklist/>, accessed: 31 Jul 2026.
