# Supplementary material for: Patient and Public Involvement and Engagement in Pediatric Health Research: A Systematic Review
Source: Health Expect. 2026 Sep 4;29(5):e70862. doi: 10.1111/hex.70862 (PMC13543670; doi:10.1111/hex.70862)
Supplement: Supplementary file 2 [file HEX-29-e70862-s002.docx]

**Appendix 2:** Search strategies for databases in the systematic review on PPIE in pediatric health research.

Pubmed:

("patient participation"[MeSH Terms] OR “stakeholder participation”[MeSH Terms:noexp] OR “community-based participatory research”[MeSH Terms] OR "patient and public involvement"[Title/Abstract] OR "patient engagement"[Title/Abstract] OR "public engagement"[Title/Abstract] OR "patient participation"[Title/Abstract] OR "patient involvement"[Title/Abstract] OR "public participation"[Title/Abstract] OR "stakeholder participation"[Title/Abstract] OR "patient empowerment"[Title/Abstract] OR "patients as partners"[Title/Abstract] OR "patient partnership in research"[Title/Abstract] OR “patient-partner engagement"[Title/Abstract] OR "patient experience and public involvement"[Title/Abstract]) AND ("biomedical research/methods"[MeSH Terms:noexp] OR “health services research”[MeSH Terms:noexp] OR “health services”[MeSH Terms:noexp] OR "clinical studies as topic/methods"[MeSH Terms] OR "health* research"[Title/Abstract] OR "medical care research"[Title/Abstract] OR "medical research"[Title/Abstract]) AND ("child"[MeSH Terms] OR "infant"[MeSH Terms] OR "child*"[Title/Abstract] OR "newborn*"[Title/Abstract] OR "congenital*"[Title/Abstract] OR "infan*"[Title/Abstract] OR "baby"[Title/Abstract] OR "babies"[Title/Abstract] OR "neonat*"[Title/Abstract] OR "pre-term"[Title/Abstract] OR "preterm*"[Title/Abstract] OR "premature birth*"[Title/Abstract] OR "NICU"[Title/Abstract] OR "preschool*"[Title/Abstract] OR "pre school*"[Title/Abstract] OR "kindergarten*"[Title/Abstract] OR "kindergarden*"[Title/Abstract] OR "elementary school*"[Title/Abstract] OR "nursery school*"[Title/Abstract] OR "schoolchild*"[Title/Abstract] OR "toddler*"[Title/Abstract] OR "boy"[Title/Abstract] OR "boys"[Title/Abstract] OR "girl*"[Title/Abstract] OR "middle school*"[Title/Abstract] OR "pubescen*"[Title/Abstract] OR "juvenile*"[Title/Abstract] OR "teen*"[Title/Abstract] OR "youth*"[Title/Abstract] OR "high school*"[Title/Abstract] OR "adolesc*"[Title/Abstract] OR "pre pubesc*"[Title/Abstract] OR "prepubesc*"[Title/Abstract] OR "adolesc*"[Title/Abstract] OR "pediat*"[Title/Abstract] OR "paediat*"[Title/Abstract]) AND (2003[dp] : 3000[dp]) AND (English[la] OR German[la])

EMBASE:

(('patient and public involvement'/de OR 'patient participation'/de) OR ((patient OR public) NEAR/3 (engagement OR participat* OR involve* OR empower* OR partner*)):ti,ab,kw) AND (('medical research'/de OR 'clinical research'/de) OR ((health* OR medical) NEAR/2 (research OR study OR studies)):ab,ti,kw) AND ('child'/de OR 'infant'/de OR 'adolescent'/de OR 'pediatrics'/de OR child*:ab,ti,kw OR newborn*:ab,ti,kw OR congenital*:ab,ti,kw OR infan*:ab,ti,kw OR baby:ab,ti,kw OR babies:ab,ti,kw OR neonat*:ab,ti,kw OR 'pre-term':ab,ti,kw OR preterm*:ab,ti,kw OR 'premature birth*':ab,ti,kw OR nicu:ab,ti,kw OR preschool*:ab,ti,kw OR 'pre school*':ab,ti,kw OR kindergarten*:ab,ti,kw OR kindergarden*:ab,ti,kw OR 'elementary school*':ab,ti,kw OR 'nursery school*':ab,ti,kw OR schoolchild*:ab,ti,kw OR toddler*:ab,ti,kw OR boy:ab,ti,kw OR boys:ab,ti,kw OR girl*:ab,ti,kw OR 'middle school*':ab,ti,kw OR pubescen*:ab,ti,kw OR juvenile*:ab,ti,kw OR teen*:ab,ti,kw OR youth*:ab,ti,kw OR 'high school*':ab,ti,kw OR adolesc*:ab,ti,kw OR 'pre-pubesc*':ab,ti,kw OR prepubesc*:ab,ti,kw OR adolesc*:ab,ti,kw OR pediat*:ab,ti,kw OR paediat*:ab,ti,kw) AND ([2003-3000]/py) AND (english:la OR german:la)

Cochrane:

(((patient OR public) NEAR/3 (engagement OR participat* OR involve* OR empower* OR partner*)):ti,ab,kw) AND (((health* OR medical) NEAR/2 (research OR study OR studies)):ab,ti,kw) AND (child*:ab,ti,kw OR newborn*:ab,ti,kw OR congenital*:ab,ti,kw OR infan*:ab,ti,kw OR baby:ab,ti,kw OR babies:ab,ti,kw OR neonat*:ab,ti,kw OR “pre-term”:ab,ti,kw OR preterm*:ab,ti,kw OR (premature NEXT birth*):ab,ti,kw OR nicu:ab,ti,kw OR preschool*:ab,ti,kw OR (pre NEXT school*):ab,ti,kw OR kindergarten*:ab,ti,kw OR kindergarden*:ab,ti,kw OR (elementary NEXT school*):ab,ti,kw OR (nursery NEXT school*):ab,ti,kw OR schoolchild*:ab,ti,kw OR toddler*:ab,ti,kw OR boy:ab,ti,kw OR boys:ab,ti,kw OR girl*:ab,ti,kw OR (middle NEXT school*):ab,ti,kw OR pubescen*:ab,ti,kw OR juvenile*:ab,ti,kw OR teen*:ab,ti,kw OR youth*:ab,ti,kw OR (high NEXT school*):ab,ti,kw OR adolesc*:ab,ti,kw OR pre-pubesc*:ab,ti,kw OR prepubesc*:ab,ti,kw OR adolesc*:ab,ti,kw OR pediat*:ab,ti,kw OR paediat*:ab,ti,kw)

PsycINFO:

(DE "Client Participation" OR TI((patient OR public) N3 (engagement OR participat* OR involve* OR empower* OR partner*)) OR AB((patient OR public OR stakeholder) N3 (engagement OR participat* OR involve* OR empower* OR partner*))) AND (( DE "Medical Sciences" OR DE "Biomedicine" ) OR TI((health* or medical) N2 (research OR study OR studies)) OR AB((health* or medical) N2 (research OR study OR studies))) AND (DE "Pediatrics" OR TI (child* OR newborn* OR congenital* OR infan* OR baby OR babies OR neonat* OR "pre-term" OR preterm* OR "premature birth*" OR NICU OR preschool* OR "pre school*" OR kindergarten* OR kindergarden* OR "elementary school*" OR "nursery school*" OR schoolchild* OR toddler* OR boy OR boys OR girl* OR "middle school*" OR pubescen* OR juvenile* OR teen* OR youth* OR "high school*" OR adolesc* OR "pre pubesc*" OR prepubesc* OR adolesc* OR pediat* OR paediat*) OR AB (child* OR newborn* OR congenital* OR infan* OR baby OR babies OR neonat* OR "pre-term" OR preterm* OR "premature birth*" OR NICU OR preschool* OR "pre school*" OR kindergarten* OR kindergarden* OR "elementary school*" OR "nursery school*" OR schoolchild* OR toddler* OR boy OR boys OR girl* OR "middle school*" OR pubescen* OR juvenile* OR teen* OR youth* OR "high school*" OR adolesc* OR "pre pubesc*" OR prepubesc* OR adolesc* OR pediat* OR paediat*))
