# Supplementary material for: Patient and Public Involvement and Engagement in Pediatric Health Research: A Systematic Review
Source: Health Expect. 2026 Sep 4;29(5):e70862. doi: 10.1111/hex.70862 (PMC13543670; doi:10.1111/hex.70862)
Supplement: Supplementary file 3 [file HEX-29-e70862-s003.docx]

**Appendix 3:** PPIE reporting quality assessment using GRIPP2 short form checklist [1] for original research articles included in the systematic review on PPIE in pediatric health research.

| **Author, Year** | **Aim** | **Methods** | **Study results** | **Discussion and conclusions** | **Reflections/critical perspective** |
| --- | --- | --- | --- | --- | --- |
| Allen, 2013 | Yes | No | No | Yes | No |
| Antal, 2017 | Yes | Yes | No | Yes | No |
| Boote, 2016 | Yes | Yes | Yes | Yes | Yes |
| Chi, 2018 | Yes | Yes | Yes | Yes | No |
| Coyne, 2024 | Yes | No | No | No | No |
| Dennehy, 2019 | Yes | Yes | Yes | Yes | Yes |
| Edwards, 2011 | Yes | Yes | Yes | Yes | Yes |
| Flegg, 2020 | Yes | Yes | No | Yes | No |
| Gignac, 2021 | Yes | Yes | Yes | Yes | Yes |
| Hall, 2019 | Yes | Yes | Yes | Yes | Yes |
| Hall, 2021 | Yes | Yes | No | Yes | Yes |
| Haynes, 2016 | Yes | Yes | Yes | Yes | No |
| Kamanda, 2013 | Yes | Yes | Yes | Yes | Yes |
| Kaufman, 2014 | Yes | Yes | Yes | Yes | Yes |
| Kearsley-Fleet, 2024 | Yes | No | No | Yes | No |
| Kendal, 2017 | Yes | Yes | Yes | Yes | Yes |
| Kim, 2020 | Yes | Yes | Yes | Yes | Yes |
| Knisley, 2021 | Yes | Yes | Yes | No | No |
| Lampa, 2022 | Yes | Yes | No | No | Yes |
| Langer, 2022 | Yes | Yes | Yes | Yes | Yes |
| Levene, 2022 | Yes | Yes | Yes | Yes | Yes |
| Lowes, 2011 | Yes | Yes | No | Yes | Yes |
| Luchtenberg, 2020 | Yes | Yes | No | Yes | Yes |
| March, 2021 | Yes | Yes | Yes | Yes | Yes |
| Mitchell, 2021 | Yes | Yes | No | Yes | Yes |
| Monk, 2019 | Yes | Yes | No | Yes | No |
| Oridota, 2023 | Yes | No | No | Yes | Yes |
| Rayment, 2017 | Yes | Yes | Yes | Yes | Yes |
| Shelef, 2016 | Yes | Yes | Yes | Yes | Yes |
| Sherratt, 2018 | Yes | Yes | Yes | Yes | Yes |
| Tume, 2016 | Yes | Yes | Yes | No | Yes |
| van Staa, 2010 | Yes | Yes | Yes | Yes | Yes |
| Vangeepuram, 2017 | Yes | No | No | Yes | No |
| Walton, 2018 | Yes | Yes | Yes | Yes | Yes |
| Warner, 2021 | Yes | Yes | No | Yes | Yes |
| Warren, 2014 | Yes | Yes | Yes | Yes | Yes |
| Watson, 2017 | Yes | Yes | Yes | Yes | Yes |

1.	Staniszewska, S., et al., *GRIPP2 reporting checklists: tools to improve reporting of patient and public involvement in research.* Bmj, 2017. **358**: p. j3453.
