# Supplementary material for: Patient and Public Involvement and Engagement in Pediatric Health Research: A Systematic Review
Source: Health Expect. 2026 Sep 4;29(5):e70862. doi: 10.1111/hex.70862 (PMC13543670; doi:10.1111/hex.70862)
Supplement: Supplementary file 4 [file HEX-29-e70862-s004.docx]

**Appendix 1:** Lay summary of systematic review on PPIE in pediatric health research.

In recent years, it has become more common for patients, families and members of the public to be directly involved in planning, conducting and disseminating health research. This approach, known as Patient and Public Involvement and Engagement (PPIE), helps make research more relevant, efficient and useful. Our study looked at how PPIE has been used in research about the health of children and young people. We systematically reviewed published studies to understand who was involved, how and when they were involved, and what benefits and challenges were reported. We looked at publications that reported how PPIE was used in 35 research projects. In the majority of them, children, adolescents or caregivers took part in PPIE activities. They were often involved in early stages of research, such as designing studies or improving strategies for the recruitment of study participants. Common benefits of these activities included better recruitment of study participants, a low number of study participants dropping out during the study, as well as personal gains for those taking part in PPIE. However, reported challenges were the need for financial and time resources and difficulties in recruiting PPIE participants. In summary, our findings from PPIE in pediatric health studies were close to those from other areas of health research. It is important that researchers follow existing guidelines for planning and reporting PPIE activities, so that experiences can be shared and improved across future studies.
